# Wall lizards display conspicuous signals to conspecifics and reduce detection by avian predators

**DOI:** 10.1093/beheco/aru126

**Published:** 2014-07-28

**Authors:** Kate L.A. Marshall, Martin Stevens

**Affiliations:** ^a^Department of Zoology, University of Cambridge, CB2 3EJ, UK and; ^b^Centre for Ecology and Conservation, University of Exeter, Penryn Campus, Penryn, Cornwall, TR10 9FE, UK

**Keywords:** camouflage, color variation, communication, signal partitioning, trade-offs, vision.

## Abstract

Visual signals are often under conflicting selection to be hidden from predators while being conspicuous to mates and rivals. Here, we investigated whether 3 different island populations of Aegean wall lizards (*Podarcis erhardii*) with variable coloration among diverse island habitats exhibit simultaneous camouflage and sexual signals. We examined whether signals appear better tuned to conspecific vision as opposed to that of avian predators, and whether background-matching camouflage and sexual signals are partitioned to specific body regions. This could facilitate both covert sexual signaling and camouflage according to the viewing perspectives of predators and conspecifics. We found that lizards typically appeared twice as conspicuous to conspecifics than to avian predators against the same visual background, largely due to lizards’ enhanced sensitivity to ultraviolet, suggesting that *P. erhardii* signals are tuned to conspecific vision to reduce detection by predators. Males were more conspicuous than females to both predators and conspecifics. In 2 populations, male backs were relatively more camouflaged to predators compared to signaling flanks, whereas in females, exposed and concealed surfaces were camouflaged to predators and generally did not differ in background matching. These findings indicate that lizard coloration evolves under the competing demands of natural and sexual selection to promote signals that are visible to conspecifics while being less perceptible to avian predators. They also elucidate how interactions between natural and sexual selection influence signal detectability and partitioning to different body regions, highlighting the importance of considering receiver vision, viewing perspectives, and signaling environments in studies of signal evolution.

## Lay Summary

Lizards and their predators see the world differently, allowing lizards (*Podarcis erhardii*) to display bright sexual signals that are less visible to hunting birds. Males are more conspicuous than females, but reduce their visibility to predators by having camouflaged backs and restricting brighter signals to their sides, which makes them less visible to birds hunting from above while still being highly visible to mates and rivals on the ground.

## INTRODUCTION

A widespread trade-off between natural and sexual selection in animals is the need for conspicuous sexual signals while minimizing detection by predators. Consequently, visual signals often reflect the competing demands of predator avoidance (camouflage) and sexual communication with conspecifics, as shown in classic work on guppies (Endler 1978, 1980). Sexual selection favors conspicuous signals because they are important in mate choice and sexual competition across a wide range of animals, predominantly in males (Andersson 1994; lizards: e.g., LeBas and Marshall 2000; Bajer et al. 2010, 2011; Pérez i de Lanuza et al. 2013a; frogs: e.g., Gomez et al. 2009; birds: e.g., Alonso-Alvarez et al. 2004; and primates: e.g., Higham et al. 2010).

However, conspicuous coloration is often costly and can increase the risk of detection by predators (e.g., Endler 1978, 1980; Stuart-Fox et al. 2003; Husak et al. 2006; but see Gōtmark 1992, 1993), particularly as predators often have visual systems tuned to detect the communication signals of their prey (Ryan et al. 1982; Robert et al., 1992; reviewed by Zuk and Kolluru 1998; Stevens 2013). In principle, animals could have several adaptations that may offset the risk of predation, including changes in behavior, communicating privately or less conspicuously within a sensory modality that predators can detect, signaling in sensory modalities that predators do not have, and partitioning of body regions for concealment and signaling according to the viewing perspective (e.g., angle) of predators and conspecifics (Endler 1992; Brandley et al. 2013; Stevens 2013). Here, we investigated whether the coloration of Aegean wall lizards (*Podarcis erhardii*) has adapted for conspicuous visual signaling to conspecifics while minimizing detection by predators through decreased conspicuousness and signal partitioning.

Research is increasingly showing that conspicuous sexual signals are located on body surfaces visible to conspecifics and less visible to predators, while camouflage is found on regions more exposed to predators (“signal partitioning”; Endler 1992). Studies on lizards, *Bicyclus* butterflies, wolf spiders, and birds have shown that dorsal body regions more exposed to birds hunting from above exhibit lower sexual dichromatism and conspicuousness, particularly with increased predation risk, whereas less exposed ventral regions more visible to conspecifics exhibit conspicuous sexual signals (Stuart-Fox and Ord 2004; Stuart-Fox et al. 2004; Oliver et al. 2009; Gluckman and Cardoso 2010; Clark et al. 2011), which can be mediated by different light conditions (Gomez and Théry 2007). However, the visual system properties of predators and conspecifics are rarely considered, and in some cases there may be important differences between their respective visual systems (for instance, in the detection of ultraviolet [UV] light and relative abundance of different photoreceptor types). Therefore, it is essential to quantify camouflage and sexual signals in a way that reflects how predators and conspecifics would perceive them in a given environment (Endler 1992; Stevens 2007, 2013). The few studies that account for both predator and conspecific visual perception have found that ventral body regions, which are less observable to predators, are more conspicuous to both conspecifics and predators, whereas more visible dorsal areas are more camouflaged to predators, particularly in sexually competing males (crabs; Cummings et al. 2008; agamid lizards; Garcia et al. 2013).

**Figure 1:**
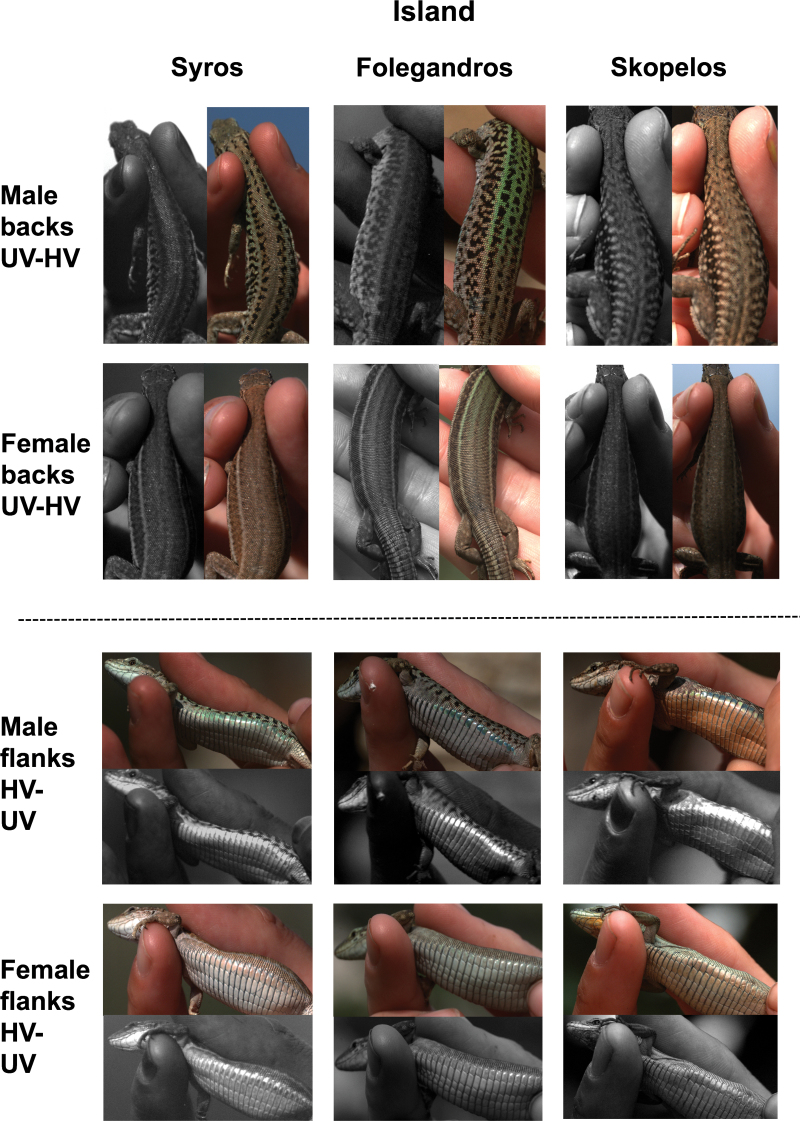
Human visible (HV) and ultraviolet (UV) images of typical male and female *Podarcis erhardii* backs and ventrolateral flanks from the 3 focal Aegean island populations (Syros, Folegandros, and Skopelos). Images: Marshall K (unpublished).

Another important consequence of the potential differences between predator and conspecific vision is that coloration could adapt under selection to be more conspicuous to conspecifics than to predators, so that sexual signals are less perceptible to potentially dangerous observers (Brandley et al. 2013). Although little is known about such communication in lizards, evidence suggests that many species have different visual sensitivities to that of their avian predators (e.g., raptors), particularly in their sensitivity to UV. This indicates that selection could promote visual signals that are more conspicuous (better tuned) to conspecific than to predator vision in lizards, as shown in work on songbird plumage coloration (Håstad et al. 2005) and UV patterning in fish (Cummings et al. 2003; Siebeck 2004; Siebeck et al. 2010). For example, many diurnal lizards are likely to have a high sensitivity to UV wavelengths, perceiving much of the UV range (300–400nm) (e.g., Loew 1994; Ellingson et al. 1995; Loew et al. 2002; Fleishman et al. 2011; Pérez i de Lanuza and Font, 2014), whereas the eyes of raptors filter out much UV light, with mainly relatively high wavelengths arriving at the retina (Lind et al. 2013). Therefore, lizards may use signals that are difficult for predators to see (e.g., shorter UV wavelengths) to potentially minimize the risk of detection (Brandley et al. 2013). However, to our knowledge it is unclear whether this occurs in lizards together with signal partitioning in variable environments. Therefore, we investigated whether 3 different island populations of *P. erhardii* that vary in coloration among their diverse island habitats and between sexes (Figure 1) use signals that are more conspicuous to conspecifics viewing them on the ground than to avian predators hunting them from above, and whether this is influenced by their varying island environments.

As previously shown in agamid lizards (Stuart-Fox and Ord 2004), *P. erhardii* may use signal partitioning and be less conspicuous to predators on islands with more open environments (e.g., dry shrubland) where the risk of detection by avian predators is high. However, on islands where the risk is relatively low (e.g., closed habitats such as forest), signal partitioning may not be so strongly favored by selection, allowing lizards to be conspicuous on all body regions, possibly to enhance sexual communication. Moreover, sexual signals may be more conspicuous on islands with shaded (darker) forest habitats to increase visibility to conspecifics, similarly to *Anolis* lizards (Leal and Fleishman 2004). Aegean island populations of *P. erhardii* are under significant risk from many visually oriented predatory birds, including several species of raptors (*Buteo* spp., *Falco* spp.) and corvids (*Corvus* spp.) that are known to be major predators of *Podarcis* and other lacertids in Europe (Martín and López 1996; Handrinos and Akriotis 1997; Castilla et al. 1999). Moreover, as in other *Podarcis* species, males experience intrasexual competition (e.g., Pérez i de Lanuza et al. 2013b; Marshall K, unpublished). Therefore, it is likely that both antipredator coloration (e.g., background-matching camouflage) and conspicuous sexual signals are present in *P. erhardii*, as in other lizards (Stuart-Fox and Ord 2004; Stuart-Fox et al. 2004; Garcia et al. 2013). Adult males exhibit ventral sexual signals that are comparable to those involved in mate acquisition and dominance signaling in other lizard species (e.g., Thompson and Moore 1991; LeBas and Marshall 2000; Whiting et al. 2006; Bajer et al. 2010, 2011; Olsson et al. 2011; Pérez i de Lanuza et al. 2013a; see Figure 1). However, little is currently known about communication traits and antipredator coloration in *P. erhardii.*

**Figure 2:**
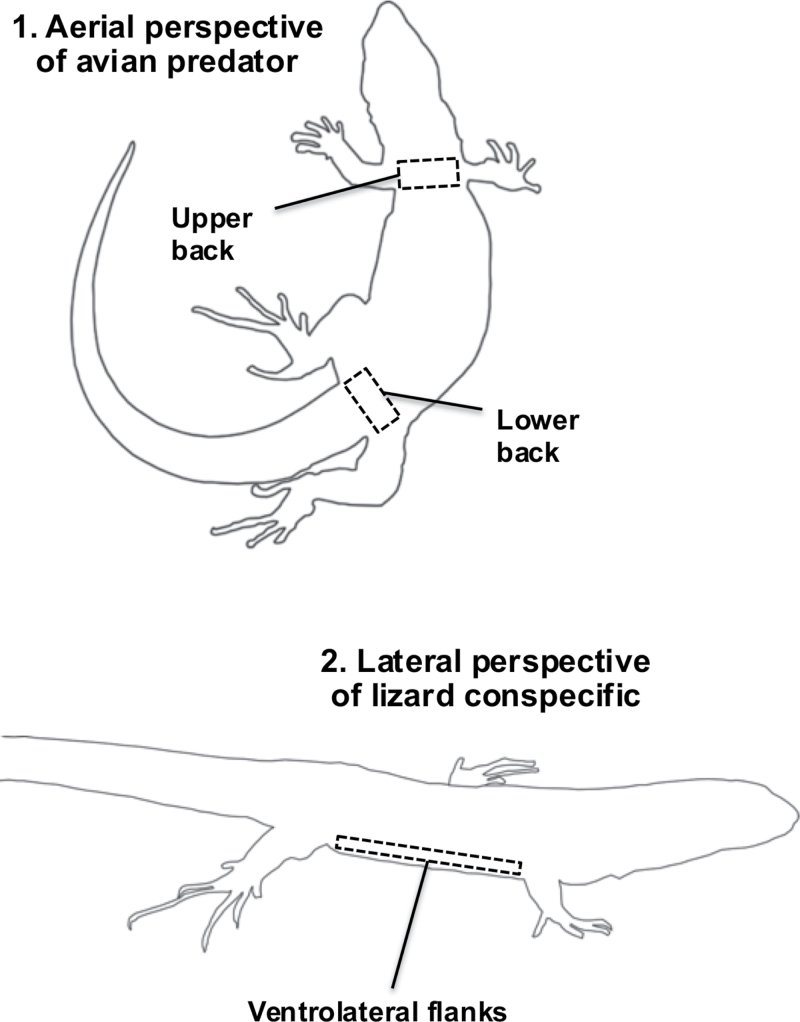
Top: an outline of an aerial view of an Aegean wall lizard (*Podarcis erhardii*) showing the body regions measured that are more visible to avian predators hunting from above (lower and upper backs). Bottom: an outline of a lateral view showing the body region measured that is potentially more visible to conspecifics on the ground, and less visible to avian predators hunting from above (ventrolateral flanks). Figure adapted from images of *P. erhardii* (Marshall K, unpublished).

We measured the conspicuousness of *P. erhardii* to avian predators and conspecifics against their corresponding natural backgrounds, and tested for differences between avian predator and conspecific perception of their coloration and conspicuousness. We investigated whether *P. erhardii* use signal partitioning by comparing perceived conspicuousness of exposed dorsal body regions (backs) and less visible ventrolateral regions (flanks). Avian predators view lizards from above, and so their more visible backs require better background matching than their less visible flanks (see Figure 2; Stuart-Fox and Ord 2004; Stuart-Fox et al. 2004; Garcia et al. 2013). Conversely, conspecifics view lizards laterally on the ground, making their flanks potentially more visible to mates and rivals (Font et al. 2009) while being less noticeable by avian predators (see Figure 2), which potentially favors more conspicuous signals to conspecifics on the flanks that are relatively hidden from avian predators. We examined how these factors differed between males and females to test for sexual dichromatism and whether different habitats and predation risk influenced conspicuousness among the 3 focal island populations.

We predicted that, due to the differing visual sensitivities of conspecifics and avian predators, both males and females would be relatively camouflaged to avian predators and more conspicuous to conspecifics. Moreover, we predicted that sexually competing males would be more conspicuous than females to both predators and conspecifics, whereas females would be relatively camouflaged (sexual dichromatism; see Figure 1). Furthermore, we predicted that females would be camouflaged on all body regions, whereas males’ backs would be relatively more camouflaged compared to their ventrolateral body regions (flanks) to minimize detection of conspicuous sexual signals by predators while still being visible to conspecifics on the ground (signal partitioning; see Figures 1 and 2). Finally, we predicted that signal conspicuousness and partitioning would differ among the 3 island populations due to different habitat light levels and potentially varying risk from avian predators (among-island variation; see Figure 1).

## MATERIALS AND METHODS

### Study sites and species

The Aegean wall lizard (*P. erhardii*) is a diurnal, small lacertid distributed across most of the South Balkans and widespread throughout many Aegean islands (Arnold and Ovenden 2002). It is listed as a species of “least concern” under the IUCN Red List classification (Lymberakis et al. 2009). We conducted field research with permission from the Greek Ministry of Environment (permit number: 166648/356) during May–August 2012 on 3 Aegean islands: Syros (37°27′ N, 24°54′ E), Folegandros (36°37′ N, 24°54′ E) and Skopelos (39°7′ N, 23°43′ E). We chose to sample lizards from these island populations because of their varying environments and risk from avian predators, which may alter lizard conspicuousness. Moreover, these islands have abundant *P. erhardii* populations and relatively accessible but remote, well-preserved natural habitats. The land used for fieldwork was publicly accessible.

### In situ photography of lizards and their backgrounds

We used digital imaging instead of spectrometry to sample coloration of lizards and their corresponding backgrounds, because it allows comprehensive color sampling, provides a way to control for natural variation in luminance intensity (shadowing) that is ignored by spectrometry, and allows non-invasive color measurements (Stevens et al. 2007). Moreover, previous research on lizard color patterns considered predator and conspecific perception of only the UV component of their signals (i.e., 300–400nm) (Garcia et al. 2013). Therefore, we measured the sensitivity of all receiver photoreceptors (300–750nm), which are important to consider as interactions among them determine how a signal is perceived through color vision (Kelber et al. 2003; Stevens and Cuthill 2007). To avoid any color fading during capture (e.g., through stress-induced decreases in body temperature; Cooper and Greenberg 1992), we photographed free-ranging lizards in situ rather than capturing them for photography. Through this method we obtained color samples of lizards and their corresponding backgrounds under the actual viewing conditions of conspecifics and avian predators.

We took images of stationary lizards and their corresponding backgrounds with a Fujifilm IS Pro UV-sensitive digital camera with a quartz CoastalOpt UV lens (Coastal Optical Systems), fitted with a UV and infrared (IR) blocking filter for photographs in the human-visible spectrum (Baader UV/IR Cut filter; transmitting between 400 and 700nm), and with a UV pass filter (Baader U filter; transmitting between 300 and 400nm) for UV images. The spectral sensitivity of our camera’s sensors had been derived prior to photography (see section 2a in the Supplementary information). We used a purpose-built filter-holder made of black opaque plastic to slide each filter onto the end of the lens when required. After the photographed lizard had fled, we took human-visible and UV images of a Spectralon^TM^ grey reflectance standard (Labsphere, Congleton, UK), which reflects light equally at 40% between 300 and 750nm, under the same light conditions as the lizard to standardize photographs for ambient light conditions. Following this “sequential method,” images of the standard were taken at the same distance, with the same camera settings, and in the same location and light conditions, as the photographed lizard (Bergman and Beehner 2008; Stevens et al. 2009).

We recorded photographed lizards’ locations using a Garmin eTrex^®^ GPS device (Schauffhausen, Switzerland) and marked it with colored tape to indicate sex and lifestage estimated using a field guide (Arnold and Ovenden 2002). In other (unpublished) work, we tested the reliability of these estimations by verifying the sex and lifestage of captive lizards, which were captured using nooses (*N* = 120). Captured males were identified by the presence of femoral pores and hemipenal bulges. Adults were identified by measurements of snout-to-vent length (SVL) using a 150mm vernier calliper (Silverline, Yeovil, UK), as adult SVL is >56 mm in the *Podarcis* genus (Pérez i de Lanuza et al. 2013a). Comparisons of estimated (from photographs) and observed sex and lifestages showed that estimations were 99% reliable (Marshall K, unpublished).

To avoid pseudoreplication, we never repeated photography in the same marked location. Furthermore, when an individual was photographed and its location marked, no other lizard was photographed within 10 m of that location. As the average home range size of lizards within the *Podarcis* genus does not typically exceed 132.2 m^2^ (Verwaijen and Van Damme 2008), it is unlikely that the same lizard would be photographed outside an area of 314 m^2^ around its original location.

### Image analysis and visual modeling

Human-visible and UV images of lizards and their backgrounds were linearized with respect to light intensity because our camera, like most others, shows a nonlinear response in image value with changes in radiance (Stevens et al. 2007; for details of the linearization process, see section 1 in the Supplementary material). Linearized images were converted to reflectance (RGB-equalized) so that RGB values in the images were independent of light conditions (see Stevens et al. 2007). Following this, and prior to visual modeling, human-visible images were aligned with their corresponding UV images in ImageJ 1.45s (64-bit) using a purpose-written script (Troscianko J, unpublished). Any images that were overexposed, could not be RGB-equalized or aligned were discarded from the analysis.

We then transformed our images to correspond to either lizard or avian predicted photon catch cone values using a mapping process based on the spectral sensitivity of our camera’s sensors (see Stevens and Cuthill 2006; Stevens et al. 2007; Pike 2011; Supplementary information, sections 2b and 2c). We converted the aligned images from camera color space to the relative photon catches of an avian and conspecific’s longwave (LW), mediumwave (MW), shortwave (SW), and UV-sensitive cone photoreceptors, using the spectral sensitivity of a peafowl (*Pavo cristatus*; Hart 2002) and a Caribbean anoline lizard (*Anolis lineatopus* (Iguanidae); Loew et al. 2002; see Supplementary information, section 2b and Supplementary Figure S2 for spectral sensitivity functions of *P. cristatus* and *A. lineatopus*). The peafowl visual system is often used as a representative of the violet-sensitive (VS) class of color vision in birds (Cuthill 2006; Hart and Hunt 2007), which is typical of the predatory birds that hunt *Podarcis* lizards and other small lacertids (i.e., raptors and corvids; Martín and López 1996; Handrinos and Akriotis 1997; Castilla et al. 1999; Ödeen and Håstad 2013). As spectral sensitivity data of lacertids is currently unavailable, we used the iguanid *A. lineatopus* as a model conspecific species, as it is one of the phylogenetically closest lizard species with available spectral data (Loew et al. 2002; Pyron et al. 2013). *A. lineatopus* and other lizards generally have a higher presence of UV receptors compared to that of avian predators (Cuthill 2006; Hart and Hunt 2007; Fleishman et al. 2011; Lind et al. 2013) and recent work shows that lacertids are capable of UV vision (Pérez i de Lanuza and Font 2014). Calibrations were performed in MATLAB v. R2011b (The MathWorks Inc. MA, USA) using self-written programs. Both calibrations were restricted to the 300–700nm range, which encompasses most of the visual spectrum of diurnal birds (Hart and Hunt 2007) and lizards (Fleishman et al. 2011).

LW, MW, SW, and UV photon catches of lizards and their corresponding backgrounds were extracted from the calibrated images in ImageJ using the selection tool. The in situ nature of the photography meant that in 9 of the images, some parts of the lizard and their background were cast in shadow. Rather than discarding useful data, we controlled for these variations in light levels by, if necessary, categorizing lizard selections into “dark” and “light” conditions in relation to the grey standard, and took similarly “dark” and “light” corresponding background selections from the same image for subsequent comparisons. Each selection area was the same in each image, but not constant across images, as selection area size depended on the size of the lizard within the image.

Backgrounds were selected based on 2 criteria: firstly, that the selection touched but did not overlap with the lizard. Secondly, to keep background type constant across images, selections were limited to rock backgrounds, avoiding areas of lichen and moss. We chose rock backgrounds because lizards were most frequently observed basking on rocks, making them potentially visible to both aerially hunting avian predators and conspecifics. The amount of background available meeting the specified selection criteria constrained how many selections could be made in each image. At least 2 background selections were taken from each image and categorized as “light” or “dark” conditions when necessary.

Lizard selections were made from 3 body regions: exposed lower and upper backs, and the ventrolateral surface (flanks) (see Figure 2). Flank selections were sometimes not viable because they were too dark due to shadow or not visible due to the angle of the photograph. Separate selections of lower and upper backs were taken because we observed color differences between these regions in many lizards. Lizard selection criteria were standardized across all images: lower back selections were taken next to the base of the tail; upper back selections were taken next to the base of the head; and flank selections were taken in the area between the hidden ventral surface and the darker ventrolateral stripe (Figure 2). Lizard selections were repeated 3 times in different areas of the focal region and averaged.

To determine whether perception of lizard conspicuousness differed between the 2 modeled visual systems, we plotted avian predator and conspecific photon catches of each of the 3 body regions and the backgrounds with which they were compared in tetrahedral color space (see Endler and Mielke 2005; Stoddard and Prum 2008).

### Background matching

To determine how well lizards matched their backgrounds as perceived by avian predators and conspecifics, we quantified color contrasts between mean photon catches of lizard body regions (flanks, lower, and upper backs) and mean photon catches of their corresponding backgrounds according to the log form of the Vorobyev and Osorio (1998) receptor noise model. To account for receptor noise, we used a Weber fraction value of 0.05 for the most frequent cone type based on data in other vertebrates (Vorobyev and Osorio 1998; Vorobyev et al. 1998). We used relative proportions of cone types in the peafowl retina to calculate avian predator-perceived chromatic contrast (LW = 0.92, MW = 1.00, SW = 0.81, UV = 0.54; Hart 2002). Because relative abundance of cone types in lizards has so far not been reliably determined, we ran 2 separate models with different scenarios of cone type abundance to calculate conspecific-perceived chromatic contrast: (a) LW = 1.00, MW = 0.33, SW = 0.33, UV = 0.33 and (b) LW = 1.00, MW = 1.00, SW = 0.33, UV = 0.33 (Fleishman L, personal communication). The results of both models were compared qualitatively to determine their reliability.

The degree of chromatic contrast generated from these models is expressed in “just-noticeable-differences” (JND). Generally, a JND of less than 1.00 indicates that 2 stimuli are indistinguishable; values between 1.00 and 3.00 should be difficult to discriminate except under optimal light conditions; and values increasing above 3.00 indicate increasingly improved discrimination (Siddiqi et al. 2004). For each image, we calculated conspecific and avian predator perception of chromatic contrast (JND) between the lizard and its corresponding background. In each (predator and conspecific) visual model, the same lizard selections were compared to the same background selections. In both visual models, the overall mean photon catch of each lizard body region (flanks, lower back, and upper back) was compared to mean photon catches of the corresponding background selections taken from the same image. The resulting JNDs were averaged to yield one JND value for each lizard region-background comparison per image (i.e., 3 JND values in total per image). There were relatively high amounts of light variation in 9 of the images, so in these cases we compared “dark” lizard regions with corresponding “dark” background regions, and “light” lizard regions with corresponding “light” background regions, to further ensure that comparisons were not distorted by varying light levels.

We quantified conspecific-perceived chromatic contrast (JND) of lizards against their backgrounds in 2 separate models with different relative abundance of cone type scenarios. The first model used scenario (a) and the second model used scenario (b) as stated above. We performed statistical analyses on data from both models and compared the results to identify any qualitative differences.

### Statistical analyses

Normality tests and residuals analysis showed that the JND data were not normally distributed. Therefore, we transformed the data to normality using a square-root transformation and used this transformed data in all statistical analyses. However, to illustrate and describe our results, we report raw (back-transformed) JND data in figures and quoted mean ± SE values. All statistical analyses were conducted in SPSS^®^ (v20).

In our statistical analyses, we tested 4 predictions. First, we analyzed whether *P. erhardii* were more conspicuous to conspecifics than to avian predators due to their different visual sensitivities. Second, we tested whether males were more conspicuous than females to both conspecifics and avian predators, caused by intrasexual competition (sexual dichromatism). Third, we tested for the presence of signal partitioning in males and females. Lizard backs are more visible than flanks to hunting birds with an aerial perspective, and flanks are more visible to conspecifics with a lateral perspective, potentially allowing covert conspicuous signals on the flanks (see Figure 2). Moreover, field observations show male *P. erhardii* flattening their flanks on the ground when threatened, presumably to enhance concealment, while they raise them off the ground when interacting with conspecifics, apparently to facilitate detection (Marshall K, unpublished). We predicted that signal partitioning would be present in *P. erhardii*, but only in males that use conspicuous sexual signals in intrasexual competition. In line with these predictions, we tested whether backs matched the background better than flanks in males, whether females were camouflaged on all body regions, and whether signal partitioning was more perceptible to conspecifics than to avian predators due to the heightened conspicuousness of signaling flanks to mates/rivals.

Fourth, we analyzed whether conspicuousness and signal partitioning varied among the 3 focal populations (Folegandros, Syros, and Skopelos) due to differing habitats and risk of detection from predatory birds (among-island variation). We tested whether lizards were less conspicuous to avian predators and use signal partitioning in the more risky, open shrubland environments of Syros. Moreover, as the relatively smaller island of Folegandros has fewer avian predators compared to Syros and Skopelos (Handrinos and Akriotis 1997), and Skopelos has a high density of closed forest environments, we tested whether signal partitioning was absent and whether lizards were more conspicuous in these populations, which experience a potentially lower risk of detection by avian predators. Finally, we tested whether sexual signals in Skopelos lizards were more conspicuous to conspecifics, possibly to increase perceptibility to mates and rivals in darker forest habitats.

To test these predictions, we conducted a mixed general linear model (GLM) that included 4 variables: island population (Folegandros, Syros, and Skopelos), sex (male and female) as between-subjects factors, body region (upper back, lower back, and flanks), and visual system perspective (lizard conspecific and avian predator) as within-subjects factors. Unless otherwise stated, we report unmodified results that assume sphericity. We additionally examined whether there were any 2-way interactions between the 4 factors. To test for any qualitative differences in the results between the relative cone abundance scenarios (a) and (b) for lizard conspecific vision, we conducted the GLM twice, first with scenario (a) and then with scenario (b).

We ensured any post hoc analyses addressed our predictions by conducting planned comparisons that did not exceed the number of experimental degrees of freedom (n−1), because these are more powerful than conservative, multiple unplanned post hoc comparisons (Ruxton and Beauchamp 2008). For each planned comparison, we reran the GLM, except that in each analysis we included only the variables selected for comparison that were relevant to testing our predictions. In all post hoc analyses, all main effects and factor interactions that were unchanged from the main GLM were not reported, and any non-significant main effects and interactions were removed and the model rerun without them.

## RESULTS

A total of 83 adult lizards was sampled from the 3 focal island populations (Folegandros = 33, Syros = 27, and Skopelos = 23; 44 males and 39 females). All 83 individuals had their lower and upper backs compared to their corresponding backgrounds, and 62 had their ventrolateral flanks compared to their corresponding backgrounds (33 males and 29 females; Syros = 22, Folegandros = 21, and Skopelos = 19). This yielded 228 lizard-background comparisons in each (predator and conspecific) visual model, totaling 456 comparisons.

There was no difference in the significance of the results of the GLM when using the relative cone abundance scenario (a) compared to scenario (b) for conspecific lizard vision, and so we only report results from scenario (a).

### Do avian predators and conspecifics perceive lizards and their backgrounds differently?

**Figure 3:**
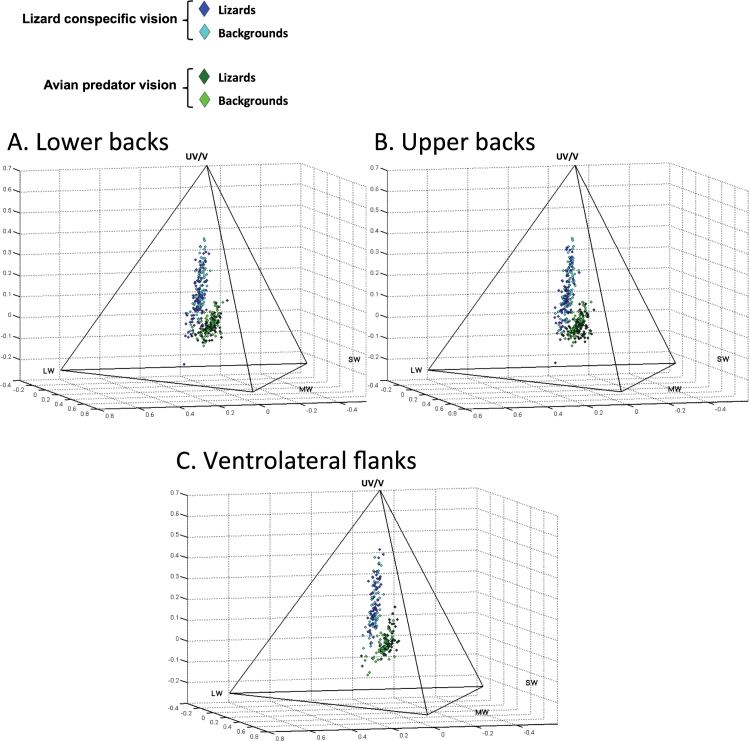
Distributions of avian- and conspecific-perceived coloration of male and female Aegean wall lizards (*Podarcis erhardii*) and their corresponding natural backgrounds in tetrahedral color space. Figures show different lizard body regions and backgrounds to which they were compared to measure their conspicuousness to avian predators and lizard conspecifics: (A) lower backs and (B) upper backs (Each *N* = 166; avian-perceived = 83, conspecific-perceived = 83) and (C) ventrolateral flanks (*N* = 124; avian perceived = 62, conspecific-perceived = 62). Alternative dark backgrounds were selected for comparison with lizard body regions cast in shadow (relative to the reflectance standard) in nine cases overall (lower backs = 5/9 cases, upper backs = 4/9 cases, flanks = 6/9 cases). Each color is a point in the tetrahedron determined by the relative stimulation of the four cone color channels, UV/V, SW, MW, and LW where the V channel refers to avian predator violet-sensitive vision and the UV channel refers to lizard conspecific ultraviolet-sensitive vision.

Plots of avian and conspecific photon catches of *P. erhardii* coloration and their corresponding natural backgrounds in tetrahedral color space showed that, in all 3 body regions (ventrolateral flanks, upper backs, and lower backs), the distribution of relative stimulation of avian predator and conspecific cones occupied distinct regions in color space (Figure 3). Specifically, relative stimulation of avian predator cones was restricted to lower UV/V regions and shifted more toward the MW/SW region. In contrast, stimulation of conspecific cones occupied a larger area of the UV/V region, extending from the same region as the avian predator UV/V distribution to a relatively higher UV/V area, revealing larger differences between lizards and their backgrounds compared to the avian predator distribution. Stimulation of conspecific cones was also shifted away from the MW/SW avian predator distribution toward the LW area (Figure 3). Moreover, although each body region occupied similar areas of color space, the distributions of ventrolateral flanks appeared more distinct from that of the backgrounds, were shifted higher in the UV/V region (especially in the relative stimulation of conspecific cones), and the distributions of avian- and conspecific-perceived coloration appeared to be more separate, compared to tetrahedral plots of the lower and upper back regions (Figure 3).

### The effects of visual system perspective, body region, sex, and island on *P.erhardii* conspicuousness

Mauchly’s test of sphericity showed that sphericity had been violated in the body region factor (*χ* ^2^_(2)_ = 16.844, *P* < 0.001), and so we report results for this factor using the Greenhouse–Geisser correction. All 4 factors had significant effects on lizard conspicuousness overall (visual system perspective, *F*_1,56_ = 90.114, *P* < 0.001, partial eta-squared [*η*_p_^2^] = 0.617; sex, *F*_1,56_ = 20.226, *P* < 0.001, *η*_p_^2^ = 0.265; body region, *F*_1.583,88.622_ = 15.565, *P* < 0.001, *η*_p_^2^ = 0.217; and island population, *F*_2,56_ = 3.486, *P* = 0.037, *η*_p_^2^ = 0.111).

Tests for 2-way interactions showed that island population significantly interacted with visual system perspective (*F*_2,56_ = 4.897, *P* = 0.011, *η*_p_^2^ = 0.149) and body region (*F*_3.165,88.622_ = 3.094, *P* = 0.029; *η*_p_^2^ = 0.099). Moreover, body region significantly interacted with visual system perspective (*F*_1.570, 87.917_ = 3.629, *P* = 0.041, *η*_p_^2^ = 0.061) and with sex (*F*_1.583,88.622_ = 6.696, *P* = 0.004, *η*_p_^2^ = 0.107). There were no other significant interactions (visual system perspective vs. sex, *F*_1,56_ = 0.582, *P* = 0.449, *η*_p_^2^ = 0.010; island vs. sex *F*_2,56_ = 2.163, *P* = 0.124, *η*_p_^2^ = 0.072). Results were interpreted from the significant interactions in planned post hoc tests, which were conducted in relation to our predictions.

### Among-island variation in avian predator versus conspecific perception of *P. erhardii* conspicuousness

As predicted, *P. erhardii* were more conspicuous to conspecifics than to avian predators in all island populations sampled (Folegandros [*F*_1, 19_ = 43.025, *P* < 0.001, *η*_p_^2^ = 0.694], conspecifics = [mean ± S.E.] 6.586±0.536 vs. avian predators = 2.955±0.223; Syros [*F*_1, 20_ = 12.612, *P* = 0.002, *η*_p_^2^ = 0.387], conspecifics = 6.441±0.454 vs. avian predators = 3.851±0.204; Skopelos [*F*_1, 17_ = 43.650, *P* < 0.001, *η*_p_^2^ = 0.720], conspecifics = 5.158±0.530 vs. avian predators = 2.613±0.252). The degree of this effect differed only between the Folegandros and Syros populations. The higher conspicuousness of *P. erhardii* to conspecific observers compared to avian predators was relatively more pronounced on Folegandros than on Syros, (*F*_1, 39_ = 5.305, *P* = 0.027, *η*_p_^2^ = 0.120).

Moreover, as predicted, *P. erhardii* were significantly more conspicuous to conspecifics than to avian predators across all body regions (flanks, [*F*_1, 56_ = 60.989, *P* < 0.001, *η*_p_^2^ = 0.521] conspecifics = 7.785±0.662 vs. avian predators = 4.050±0.340; upper backs, [*F*_1, 77_ = 46.421, *P* < 0.001, *η*_p_^2^ = 0.376], conspecifics = 5.724±0.451 vs. avian predators = 2.940±0.173; lower backs, [*F*_1, 77_ = 62.654, *P* < 0.001, *η*_p_^2^ = 0.449], conspecifics = 5.300±0.437 vs. avian predators = 2.705±0.180). The degree of this effect was no different between lower backs and flanks (*F*_1, 56_ = 2.399, *P* = 0.127, *η*_p_^2^ = 0.041) and between lower and upper backs (*F*_1, 56_ = 0.090, *P* = 0.765, *η*_p_^2^ = 0.001), however the higher conspicuousness to conspecifics was relatively reduced on the upper back region compared to the flanks (*F*_1, 56_ = 5.474, *P* = 0.023, *η*_p_^2^ = 0.089).

### Signal partitioning and sexual dichromatism

Males were more conspicuous than females on the flanks and upper backs, but not on the lower back region (flanks [*F*_1, 56_ = 33.911, *P* < 0.001, *η*_p_^2^ = 0.377], male = 7.920±0.579 vs. female = 3.639±0.398; upper backs [*F*_1, 77_ = 12.166, *P* = 0.001, *η*_p_^2^ = 0.136], male = 5.285±0.406 vs. female = 3.257±0.282; lower backs [*F*_1, 77_ = 2.266, *P* = 0.136, *η*_p_^2^ = 0.029], male = 4.548±0.391 vs. female = 3.388±0.309), suggesting that both flanks and upper backs are sexually dichromatic signals in *P. erhardii*.

As predicted, in females there was no difference in conspicuousness between upper/lower backs and flanks, whereas in males, flanks were significantly more conspicuous than both upper and lower backs (females: lower backs vs. flanks, *F*_1, 26_ = 0.542, *P* = 0.468, *η*_p_^2^ = 0.020; upper backs vs. flanks (*F*_1, 26_ = 1.468, *P* = 0.237, *η*_p_^2^ = 0.053). Males: flanks versus upper backs, *F*_1, 30_ = 32.518, *P* < 0.001, *η*_p_^2^ = 0.520; flanks versus lower backs, *F*_1, 30_ = 32.478, *P* < 0.001, *η*_p_^2^ = 0.520).

### Among-island variation in signal partitioning

Flanks were more conspicuous than lower backs in both the Syros and Skopelos populations. However, in Folegandros lizards flanks and lower backs were no different in conspicuousness (Syros [*F*_1, 20_ = 14.916, *P* = 0.001, *η*_p_^2^ = 0.427], flanks = 6.697±0.538 vs. lower backs = 4.215±0.370; Skopelos [*F*_1, 17_ = 16.621, *P* = 0.001, *η*_p_^2^ = 0.494], flanks = 5.788±0.813 vs. lower backs = 3.122±0.334; Folegandros [*F*_1, 19_ = 0.042, *P* = 0.839, *η*_p_^2^ = 0.002], flanks = 5.218±0.763 vs. lower backs = 4.443±0.512).

Flanks were also more conspicuous than upper backs in both the Syros and Skopelos populations, however again this effect was not found in Folegandros lizards (Syros [*F*_1, 20_ = 13.527, *P* = 0.001, *η*_p_^2^ = 0.403], flanks = 6.697±0.538 vs. upper backs = 4.812±0.445; Skopelos [*F*_1, 17_ = 16.881, *P* = 0.001, *η*_p_^2^ = 0.498], flanks = 5.788±0.813 vs. upper backs = 3.078±0.374; Folegandros, *F*_1, 19_ = 0.364, *P* = 0.554, *η*_p_^2^ = 0.019, flanks = 5.218±0.763 vs. upper backs = 4.813±0.471).

In the Skopelos population, the higher conspicuousness of flanks compared to lower and upper backs was found only in males (flanks vs. lower backs [*F*_1, 17_ = 18.513, *P* < 0.001, *η*_p_^2^ = 0.521]; flanks vs. upper backs [*F*_1, 17_ = 19.868, *P* < 0.001, *η*_p_^2^ = 0.539]. Males: flanks = 8.638±1.223; lower backs = 2.857±0.533; upper backs = 2.579±0.590. Females: flanks = 3.223±0.715; lower backs = 3.325±0.430; upper backs = 3.461±0.478). Both conspecifics and avian predators perceived Skopelos male flanks as more conspicuous than their upper and lower backs (conspecific vision: flanks vs. upper backs [*F*_1, 8_ = 54.597, *P* < 0.001, *η*_p_^2^ = 0.872], flanks vs. lower backs [*F*_1, 8_ = 61.700, *P* < 0.001, *η*_p_^2^ = 0.885], flanks = 11.430±1.885; lower backs = 3.803±0.889; upper backs = 3.281±1.092. Avian predator vision: flanks vs. upper backs [*F*_1, 8_ = 22.833, *P* = 0.001, *η*_p_^2^ = 0.741]; flanks vs. lower backs [*F*_1, 8_ = 29.529, *P* = 0.001, *η*_p_^2^ = 0.787]; flanks = 5.847±0.923, lower backs = 1.912±0.456, upper backs = 1.877±0.408). Significant interactions showed that these effects were more pronounced to conspecific observers compared to avian predators (flanks vs. upper backs [*F*_1, 8_ = 7.722, *P* = 0.024, *η*_p_^2^ = 0.491]; flanks vs. lower backs [*F*_1, 8_ = 7.474, *P* = 0.026, *η*_p_^2^ = 0.483]).

Conversely, in the Syros population, both males and females were more conspicuous on their flanks relative to their lower and upper backs (flanks vs. lower backs [*F*_1, 20_ = 0.015, *P* = 0.903, *η*_p_^2^ = 0.001], flanks vs. upper backs [*F*_1, 20_ = 4.003, *P* = 0.059, *η*_p_^2^ = 0.167]. Females: flanks = 4.988±0.532, lower backs = 3.509±0.596, upper backs = 2.696±0.513. Males: flanks = 7.338±0.683, lower backs = 4.567±0.463, upper backs = 5.872±0.538). The extent of this difference in conspicuousness between Syros lizards’ flanks and lower backs did not vary between conspecific and avian predators (*F*_1, 20_ = 0.718, *P* = 0.407, *η*_p_^2^ = 0.035; conspecific vision: flanks = 8.341±0.877 vs. lower backs = 5.333±0.612. Avian predator vision: flanks = 5.053±0.401 vs. lower backs = 3.097±0.298). However, a significant interaction between Syros lizard body region (upper backs and flanks) and visual system perspective showed that the higher conspicuousness of flanks compared to upper backs was more pronounced to conspecific observers than to avian predators, ([*F*_1, 20_ = 4.758, *P* = 0.041, *η*_p_^2^ = 0.192]. Conspecifics: flanks = 8.341±0.877 vs. upper backs = 6.001±0.790. Avian predators: flanks = 5.053±0.401 vs. upper backs = 3.625±0.270).

In the Folegandros population, there were no differences in the conspicuousness of lower/upper backs and flanks in both males and females (flanks vs. lower backs [*F*_1, 19_ = 1.222, *P* = 0.283, *η*_p_^2^ = 0.060]; flanks vs. upper backs [*F*_1, 19_ = 1.664, *P* = 0.283, *η*_p_^2^ = 0.060]. Males: flanks = 8.276±1.437; lower backs = 5.582±0.840; upper backs = 6.317±0.759. Females: flanks = 3.336±0.637; lower backs = 3.371±0.553; upper backs = 3.398±0.464). Moreover, the perceived differences in conspicuousness between Folegandros lizards’ flanks and upper/lower backs was no different between conspecific and avian predator observers (flanks vs. lower backs [*F*_1, 19_ = 0.048, *P* = 0.829, *η*_p_^2^ = 0.003]; flanks vs. upper backs [*F*_1, 19_ = 0.080, *P* = 0.780, *η*_p_^2^ = 0.004]; Conspecifics: flanks = 7.218±1.249; lower backs = 6.144±0.884; upper backs = 6.626±0.786. Avian predators: flanks = 3.219±0.652; lower backs = 2.742±0.318; upper backs = 3.001±0.282).

## DISCUSSION

**Figure 4:**
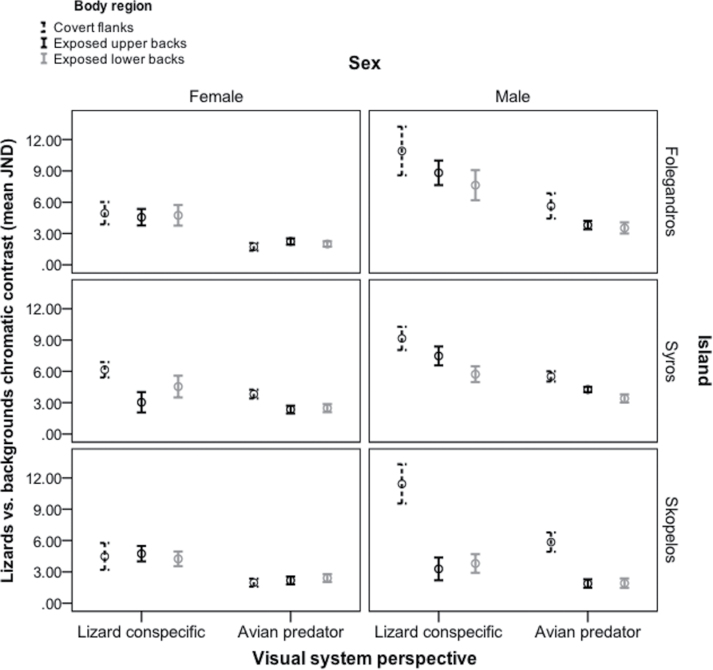
Interval plots showing modeled avian and conspecific perception of chromatic contrast (JND) of exposed dorsal (upper and lower backs) and covert ventrolateral flanks of male and female Aegean wall lizards (*Podarcis erhardii*) against corresponding backgrounds on 3 focal Aegean islands: Folegandros (*N* = 33; 17 females, 16 males; flanks measured in 13 females, 8 males), Syros (*N* = 27; 9 females, 18 males; flanks measured in 6 females, 16 males) and Skopelos (*N* = 23; 13 females, 10 males; flanks measured in 10 females, 9 males). Error bars represent ±1 SE. Values >3.00 JND denote an increasing ability to discriminate lizards from the background, whereas values ≤3.00 JND denote lizard coloration generally indistinguishable from the background.

Our results show that the variable coloration of 3 island populations of Aegean wall lizards (*P. erhardii*), which inhabit diverse island environments, has evolved under the conflicting demands of natural and sexual selection. Initially, as demonstrated in previous research (Stuart-Fox and Ord 2004; Stuart-Fox et al. 2004; Cummings et al. 2008; Pérez i de Lanuza et al. 2013a), our findings indicate sexual dichromatism. Males were more conspicuous than females on the flanks and upper backs, and females were relatively camouflaged on all body regions (Figure 4). This suggests that concealing coloration has been favored in females whereas conspicuous signals on specific body regions is more important in males as a consequence of intrasexual competition. Moreover, in line with previous work (e.g., Endler 1992; Oliver et al. 2009; Clark et al. 2011; Garcia et al. 2013), we found some evidence of signal partitioning, as more exposed body regions were better camouflaged compared to covert body regions on 2 islands (Skopelos and Syros; Figure 4). On all 3 islands and body regions, both males and females appeared more conspicuous to conspecifics than to avian predators against the same visual background (Figure 4), which was largely caused by differences in their color perception of *P. erhardii* signals (Figure 3). This indicates that *P. erhardii* use signals that are better tuned to the visual systems of their conspecifics than to that of their avian predators (Brandley et al. 2013).

### Increased conspicuousness to conspecifics in *P. erhardii*

**Figure 5:**
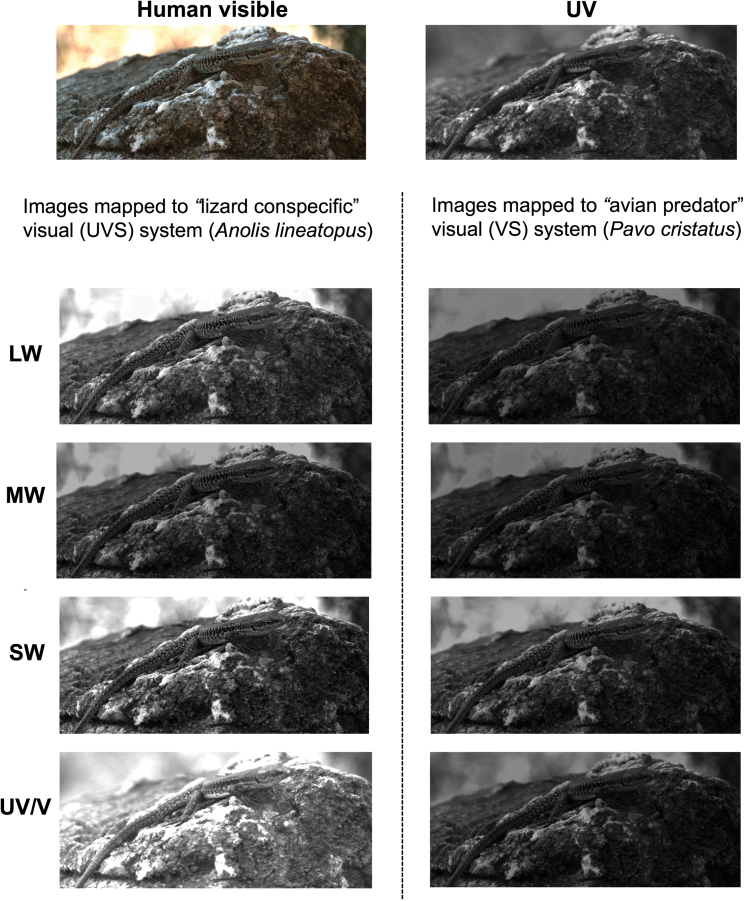
An example of in situ human visible and ultraviolet (UV) photographs of a male Aegean wall lizard (*Podarcis erhardii*) on Skopelos island, mapped to the sensitivity of the LW, MW, SW, and UV/V photoreceptors of an “avian predator” violet-sensitive (VS) visual system (peafowl, *Pavo cristatus*) and a “lizard conspecific” ultraviolet-sensitive (UVS) visual system (*Anolis lineatopus*). Note the relatively more conspicuous lizard-mapped images compared to the avian-mapped images. Images: Marshall K (unpublished).

The increased conspicuousness of *P. erhardii* to conspecifics compared to avian predators potentially enhances female camouflage and allows males to use conspicuous sexual signals that minimize the risk of detection by predators (Endler 1980; Brandley et al. 2013; Stevens 2013). Tetrahedral color plots illustrate differences between conspecific and avian perception of *P. erhardii* coloration, which are primarily driven by lizards’ relatively higher sensitivity to UV and red color signals compared to avian predators’ lower sensitivity to UV and heightened perception of blue–green colors (Figure 3). An image of *P. erhardii* mapped to conspecific and avian predator vision further demonstrates these differences, particularly in the UV channel (Figure 5). These findings are in line with evidence indicating that avian predators, such as raptors, have different visual sensitivities to lizards, especially in their sensitivity to UV (Cuthill 2006; Hart and Hunt 2007).

Our model conspecific lizard visual system (*A. lineatopus* [Iguanidae]; Loew et al. 2002), which belongs to one of the phylogenetically closest lizard families to Lacertidae with available spectral data (Pyron et al. 2013), has a higher presence of UV receptors compared that of our model avian predator visual system (*P. cristatus*). This model avian predator species is congruent with the vision of predatory raptors (*Buteo* spp., *Falco* spp.) and corvids (*Corvus* spp.) that potentially prey on *P. erhardii* (Martín and López 1996; Handrinos and Akriotis 1997; Castilla et al. 1999), as these birds have a violet-sensitive (VS) visual system with a lower sensitivity to UV (Hart 2002; Cuthill 2006; Ödeen and Håstad 2013). Moreover, recent research has shown that raptor eyes filter out much UV light, with predominantly relatively long wavelengths arriving at the retina (Lind et al. 2013), whereas lacertids are capable of UV vision as their ocular media transmit shorter wavelengths down to 300nm, and behavioral tests show that they can discriminate between the presence/absence of UV (Pérez i de Lanuza and Font 2014). Therefore, our findings indicate that *P. erhardii* have evolved sexual signals that are better tuned to the visual sensitivities of their mates and rivals compared to that of their avian predators.

Although no studies to our knowledge provide behavioral evidence showing that lizard signals are better tuned to conspecific than to predator vision, many other species show reflectance peaks in the UV range that may function as UV signals less detectable by the visual systems of avian predators (Fleishman et al. 1993, 2011; Pérez i de Lanuza et al. 2013a). Research on visual communication in fish has found convincing evidence that the UV waveband is used as a “private” communication channel that is imperceptible to predators, for instance in the complex UV facial patterns of Ambon damselfish (*Pomacentrus amboinensis*) (Cummings et al. 2003; Siebeck 2004; Siebeck et al. 2010). However, as *P. erhardii* signals are reduced but still perceptible to predators (i.e., >1 JND), we cannot conclude that *P. erhardii* use such “private” signals in this study. Nonetheless, it is well known that UV signals are important in lizard intra- and intersexual communication (e.g., LeBas and Marshall 2000; Stapley and Whiting 2006; Whiting et al. 2006; Bajer et al. 2010, 2011), indicating that *P. erhardii* use signals that are better tuned to conspecific vision than to predator vision for relatively covert sexual communication, although behavioral experiments are needed to determine their exact role. This may signify a coevolutionary phase in which *P. erhardii* are evolving private visual signals to eventually prevent detection by avian predators altogether, or conversely, that avian predators have started to better detect lizard coloration that once functioned as a private channel of communication.

### Signal partitioning in *P. erhardii* males

Our results also indicate that camouflage and sexual signals in 2 island populations of *P. erhardii* have partitioned to different body regions depending on the visual perspectives of avian predators and conspecifics (Endler 1992). In line with our predictions, signal partitioning appears to be found in males of 2 populations, which is probably due to high levels of intrasexual competition favoring conspicuous sexual signals. This potentially allows effective transmission of conspicuous sexual signals to mates and rivals on the ground while minimizing detection by avian predators hunting from above. Specifically, in Syros and Skopelos males, exposed backs were relatively camouflaged compared to ventrolateral flanks, which are less visible to avian predators (Figures 2 and 4). These findings replicate previous results in other lizards, as well as wolf spiders, *Bicyclus* butterflies, birds, and crabs (Stuart-Fox and Ord 2004; Stuart-Fox et al. 2004; Gomez and Théry 2007; Cummings et al. 2008; Oliver et al. 2009; Gluckman and Cardoso 2010; Clark et al. 2011; Garcia et al. 2013), showing that body regions hidden from a predator’s viewing perspective but more observable to a conspecific’s perspective are more conspicuous in males, whereas body regions more observable to a predator’s perspective are more camouflaged (see Figure 2). Moreover, we further show that differences in conspicuousness between flanks and backs are more detectable by conspecifics compared to avian predators. Taken together, these results suggest that some Aegean populations of male *P. erhardii* partition signals that are better tuned to conspecific vision than to avian predator vision, which may be complementary adaptations to enhance concealment of conspicuous sexual signals from predators.

### Among-island variation of signal partitioning and conspicuousness in *P. erhardii*

Across all 3 island populations sampled, both males and females were consistently more conspicuous to conspecifics than to avian predators (particularly in Folegandros lizards) and males were more conspicuous than females on the flanks and upper backs. The consistency of these findings across 3 distinct island populations with varying environments strengthens our conclusions that *P. erhardii* are sexually dichromatic and use signals that are less detectable by avian predators. This further predicts that optimizing camouflage and/or sexual signaling in varying local environments (local adaptation) has caused color variation in *P. erhardii*, as found in other lizards (e.g., Leal and Fleishman 2004; Stuart-Fox et al. 2004; Robertson and Rosenblum 2009). Importantly, this can potentially lead to reproductive isolation and speciation, as shown in African cichlid fish (Seehausen et al. 2008). However, it is important to note that the consistency of these results may have arisen through similarities among the different island backgrounds and lizard populations, rather than local adaptation. This will be addressed in a companion paper exploring the possible role of local adaptation in causing color variation among different island populations of *P. erhardii* (Marshall K, unpublished).

Our findings also suggest that adaptation to different island environments with varying risk from avian predators influences signal partitioning. The presence of signal partitioning in Syros lizards occupying risky open habitats with many avian predators, and its absence in the Folegandros population, which is threatened by fewer avian predators (Handrinos and Akriotis 1997), supported our predictions based on previous work given that signal partitioning is more likely to evolve in habitats where there is a potentially higher cost of signaling on exposed body regions (e.g., Endler 1992; Stuart-Fox and Ord 2004). Buzzards (*Buteo buteo*), ravens (*Corvus corax*), and carrion crows (*Corvus corone*) typically prey on lizards, but are not found on the relatively smaller island of Folegandros and are present on Syros and Skopelos, along with several other avian predator species (Handrinos and Akriotis 1997). Therefore, Folegandros males may be able to afford to be relatively conspicuous on all body regions due to the potentially lower risk of detection by avian predators, possibly allowing enhanced visual communication with conspecifics. However, compared to the Syros population, Folegandros lizards use signals that are relatively better tuned to be conspicuous to conspecifics and camouflaged to avian predators. This may be a way of compensating for the absence of signal partitioning in their open shrubland habitats, which still pose some risk from avian predators, while gaining the reproductive advantages of being relatively conspicuous on all body regions.

Conversely, Syros lizards use signal partitioning together with signals that are more visible to conspecifics than to avian predators. Combining these strategies may help to avoid detection in the relatively more risky environments of Syros. However, this raises the question: if Syros lizards are under such high risk from predators, then why are their signals not as well tuned to avoiding detection by predators compared to Folegandros lizards? We suggest that some additional cost constrains signals from becoming optimally tuned to the visual systems of potential observers on Syros (e.g., color signals that facilitate thermoregulation; Geen and Johnston 2014; Reguera et al. 2014), although this requires further investigation.

An unexpected finding was that Syros females appear to use signal partitioning as well as males, despite our assumption based on previous work that only males would need conspicuous covert signals for sexual competition and mate acquisition (e.g., LeBas and Marshall 2000; Stuart-Fox and Ord 2004; Stuart-Fox et al. 2004; Cummings et al. 2008; Bajer et al. 2010, 2011). Signal partitioning in Syros females may be caused by the relatively high population density on the island and the ensuing high level of sexual competition, which may require females to use signals to compete for and/or attract the best males (Marshall K, personal observation). Sexual signals are not unusual in female lizards, such as in stimulating male courtship and attracting males (e.g., LeBas and Marshall 2000; Baird 2004; Weiss 2006) and are likely to evolve under high levels of sexual competition, such as that found on Syros. Certainly, the flanks of Syros females appear to be more conspicuous than that of females on other islands (to human vision) (Figure 1). However, further behavioral tests are needed to determine whether *P. erhardii* females use conspecific-perceived sexual signals, and how this differs among islands with varying levels of sexual competition.

Another unexpected result was the presence of signal partitioning in male populations occupying more closed and thus potentially less risky forest environments on Skopelos. These findings are incongruent with our predictions based on previous studies (e.g., Endler 1992; Stuart-Fox and Ord 2004). Signal partitioning may have evolved in Skopelos males to reduce detection by resident avian predators known to hunt in closed forest environments (e.g., sparrowhawks, *Accipiter nisus*; Marquiss and Newton 1982; Handrinos and Akriotis 1997; Selås and Rafoss 2008). Certainly, their well-camouflaged backs may counteract any increased risk caused by having conspicuous flanks. Moreover, Skopelos lizards may need to leave forested habitats to forage and/or search for mates in more risky, open meadow environments that are also found on the island (where many lizards in the current study were sampled). Under these conditions, signal partitioning in Skopelos males may facilitate reduced risk from predators across different types of habitat patches, although this would require their dorsal camouflage to match a wide variety of backgrounds (Merilaita et al. 1999; Houston et al. 2010). Therefore, a valuable aim in future work would be to test the conspicuousness of backs relative to the flanks in individual lizards, both within- and among-island populations.

Another explanation for the unexpected presence of signal partitioning in Skopelos males is the darker environments created by the high density of deep-shaded pine forests on the island, particularly as previous studies report that variation in habitat light levels affects signal partitioning and detectability (e.g., *Anolis* lizards; Leal and Fleishman 2004; birds; Gomez and Théry 2007). Brighter sexual signals relative to backs may increase perceptibility to conspecifics in darker environments (Endler 1992; Leal and Fleishman 2004; see Figure 5), while the high amount of vegetation cover could serve as a visual barrier to prevent detection by predators.

In summary, we have shown that coloration in lizards enables simultaneous conspicuous sexual signaling and camouflage by partitioning signals that are better tuned to the visual systems of their conspecifics than to that of their avian predators. This indicates that the conflicting demands of natural and sexual selection can affect both the detectability of signals by different receivers and signal location on the body. Future work should consider how other adaptations (e.g., antipredator behavior and/or signaling in other sensory modalities that are undetectable by predators, such as chemical signals in the form of scent marks) might also help to reduce risk of detection by predators. These results also emphasize the importance of quantifying signal conspicuousness in relation to natural signaling backgrounds and environments, such as varying habitat light levels and potential risk from predators, and of considering both the different viewing perspectives and the visual system properties of predators and conspecifics, which are factors rarely considered together in studies of signal evolution.

## SUPPLEMENTARY MATERIAL

Supplementary material can be found at http://www.beheco.oxfordjournals.org/

## FUNDING

This work was supported by a Biotechnology and Biological Sciences Research Council studentship and Magdalene College, Cambridge (K.L.A.M), and a Biotechnology and Biological Sciences Research Council and David Philips Research Fellowship (BB/G022887/1) to M.S.

## Supplementary Material

Supplementary Data
