# Supplementary material for: Wall lizards display conspicuous signals to conspecifics and reduce detection by avian predators
Source: Behav Ecol. 2014 Jul 28;25(6):1325–37. doi: 10.1093/beheco/aru126 (PMC4235580; doi:10.1093/beheco/aru126)
Supplement: Supplementary Data [file supp_25_6_1325__index.html]

Wall lizards display conspicuous signals to conspecifics and reduce detection by avian predators — Supplementary Data 

# Wall lizards display conspicuous signals to conspecifics and reduce detection by avian predators

## Supplementary Data

Data files

**Files in this Data Supplement:**

- Supplementary Data - Supplementary Data
