# Supplementary material for: Wall lizards display conspicuous signals to conspecifics and reduce detection by avian predators
Source: Behav Ecol. 2014 Jul 28;25(6):1325–37. doi: 10.1093/beheco/aru126 (PMC4235580; doi:10.1093/beheco/aru126)
Supplement: Supplementary Data [file supp_aru126_Supplementary_Information.docx]

**Wall lizards display conspicuous signals to conspecifics and reduce detection by avian predators**

**Supplementary Information**

**1. Linearization process and illumination conditions for calibrating images**

Nearly all cameras show a non-linear response in image value to changes in light levels that needs linearizing before accurate data can be obtained (e.g. Westland & Ripamonti, 2004; Stevens et al. 2007; Garcia et al. 2013, 2014). As with past work (e.g. Stevens et al. 2014), we calculated the response of our camera (in terms of image pixel values) to a set of eight Spectralon grey standards with varying reflectance values between 2-99% and wrote a linearization file for MATLAB. Previous tests of this and similar linearization processes under a range of light conditions have found them to be highly robust (Stevens et al. 2007; Pike, 2011; Garcia et al. 2014). The linearization process we used in the present study has been found to be extremely accurate, with R^2^ values always > 0.99 for each image channel.

**2. Camera to animal colour space mapping procedure**

Cameras are increasingly being used to study animal coloration, and tests show that they can reliably be used to convert coloration to receiver (predator and conspecific) vision (e.g. Párraga et al. 2002; Stevens & Cuthill 2006; Pike 2011; Stevens et al. 2014). The key to this procedure is that, rather than analysing similarities between camera and animal spectral sensitivities, it converts coloration from camera colour space to animal colour space based on the spectral sensitivities of both systems (i.e. the relative sensitivity of the camera’s sensors and the animal’s photoreceptors). This ‘mapping’ procedure has been extensively described elsewhere (see Westland & Ripamonti, 2004; Pike, 2011; Stevens et al. 2007; Stevens & Cuthill 2006) so we do not repeat the detail here. Below we outline the key steps we used to map images of *Podarcis erhardii* to animal colour space, involving a) derivation of the spectral sensitivity of our camera, b) mapping procedure and c) verifying the accuracy of the mapping procedure.

**a) Derivation of camera sensitivity functions**

The first step involved quantifying the spectral sensitivity of our camera’s sensors. Here, we used a combination of old and new techniques, the latter that we have recently developed in our lab (J Troscianko & M Stevens, in prep). Older approaches have worked by taking photographs of a standard object through a set of interference bandpass filters combined with measurements of irradiance (e.g. Párraga et al. 2002; Stevens & Cuthill 2006), or by calculating sensitivity curves based on taking photographs of a set of coloured standards and using a quadratic programming procedure to estimate curves (Pike, 2011). These two approaches show a close correspondence with each other (Pike, 2011), and with our new procedure.

Our new method essentially converts the camera into a spectrophotometer by placing a pair of dispersing prisms or a diffraction grating between the lens elements and camera sensor. The lens (and any desired filters) was arranged so that they focused a narrow beam of full-spectrum light on to the camera sensor. Calibration of wavelength locations on the sensor was achieved by using a light source that has peaks at known locations. Calibration of the camera's sensitivity to these known wavelengths was then calculated (Figure S1). The diffraction grating methodology to calculate camera sensitivity has an advantage over the Pike method in that it requires no prior information about the shape (for example, number of peaks) in the camera's sensitivity functions.


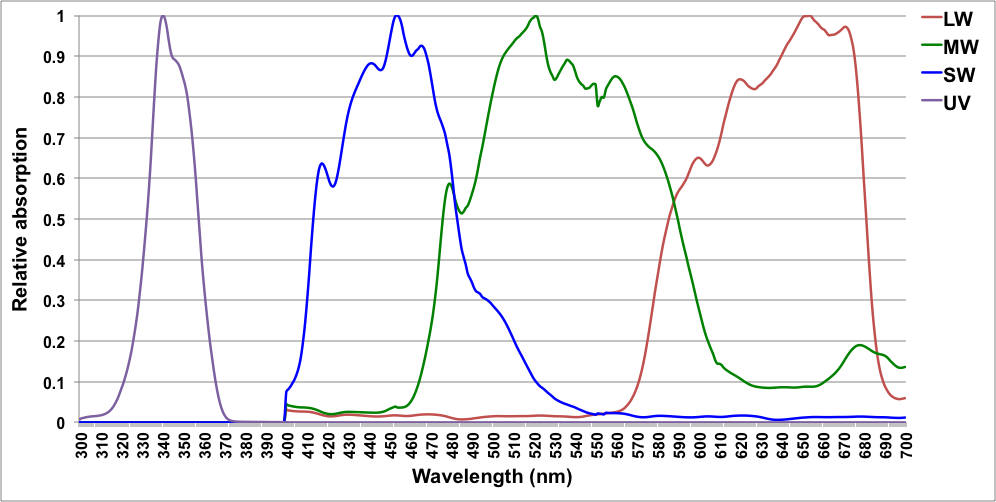


Figure S1: Showing (normalized, max value=1) spectral sensitivity of the LW, MW, SW and UV sensors of the Fuji ISPro ISO400 camera used to photograph images of *Podarcis erhardii* for subsequent mapping to animal colour space.

**b) Mapping from camera colour space to animal colour space**

Once we had obtained the spectral sensitivity of our camera’s sensors, we were then able to implement the mapping procedure. This followed previous methods we, and others, have used based on a polynomial equation (Westland & Ripamonti, 2004; Stevens & Cuthill 2006; Stevens et al. 2007; Pike, 2011; Stevens et al. 2014). Here, our model is generated to convert to animal cone-catch quanta based on a dataset of >3000 natural spectra, and converted from camera colour space to peafowl *Pavo cristatus* VS (“avian predator”) and to *Anolis lineatopus* (“lizard conspecific”) colour space, using the spectral sensitivities of their photoreceptors (Figure S2). An example of an in situ image of *P. erhardii* mapped to peafowl and *A. lineatopus* colour space is shown in Figure 5 in the main manuscript.

**A) Peafowl (*Pavo cristatus*)**
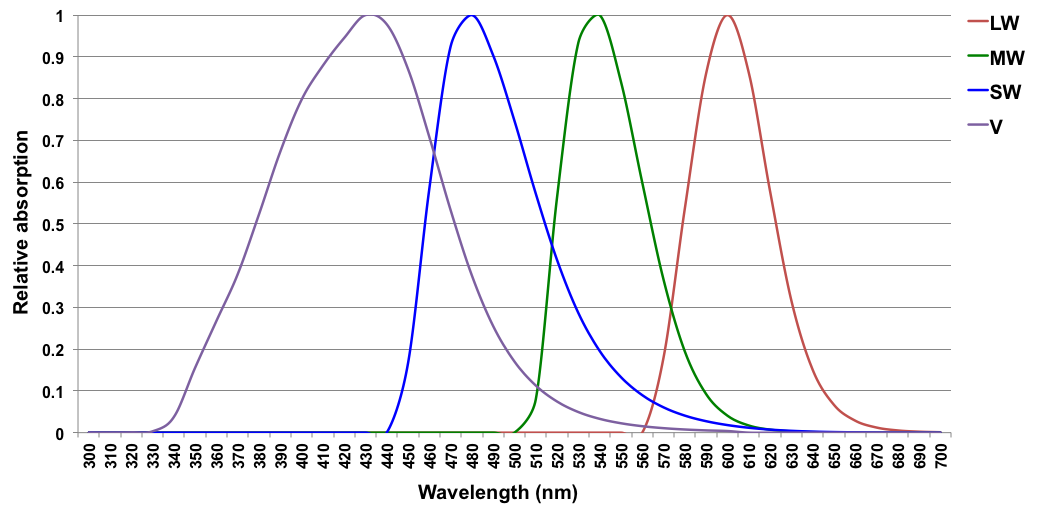


**B) *Anolis lineatopus***


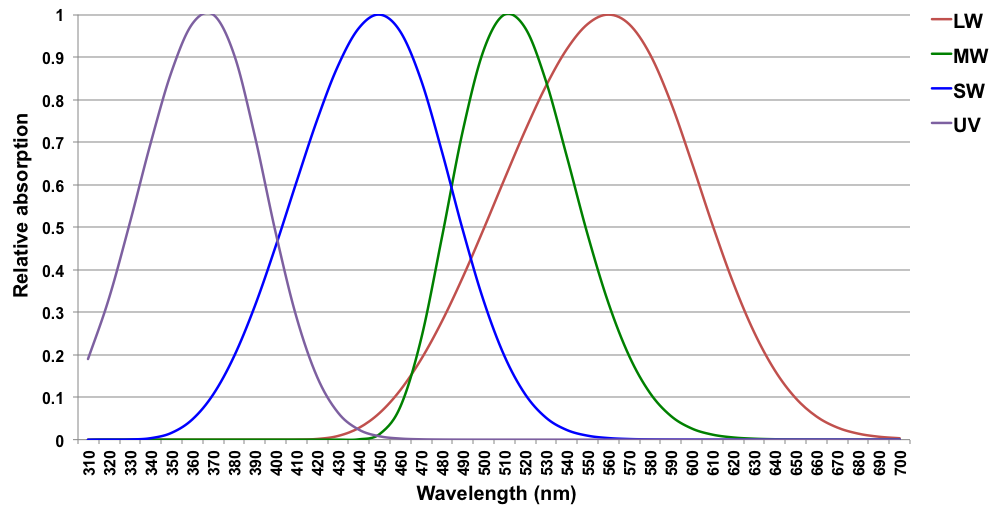


Figure S2: Showing (normalized, max value=1) spectral sensitivity of the LW, MW, SW and UV/V photoreceptors of A) peafowl (*Pavo cristatus*) (Hart, 2002) and B) a Caribbean anoline lizard (*Anolis lineatopus*) (Loew et al. 2002) used to map *Podarcis erhardii* coloration from camera (Fuji ISPro) to *P. cristatus* (“avian predator”) and to *A. lineatopus* (“lizard conspecific”) colour space.

**c) The accuracy of the mapping procedure**

We have tested the accuracy of this mapping technique for a number of cameras by comparing camera-based cone-catch estimates for a given species (e.g. peafowl) with spectrophotometer-based cone-catch estimates of a colour-chart to verify accuracy. We find R^2^ values for the channels ranging from 0.96 to 0.98 – i.e. extremely close in predicted cone catch values to those obtained with a spectrometer, with the latter also likely to have other sources of error associated (Stevens et al. 2014). Earlier work has also shown the mapping technique to be highly accurate (Stevens & Cuthill, 2006; Pike, 2011).

We note that the above methods of calculating camera sensitivity curves also include the transmission of the filters (UV blocking for the RGB, and UV pass for the UV, of which the red sensor is most sensitive) and the lens (which transmits UV light).

**References**

Garcia JE, Rohr D, Dyer AG. 2013. Trade-off between camouflage and sexual dimorphism revealed by UV digital imaging: the case of Australian Mallee dragons (*Ctenophorus fordi*). J Exp Biol. 216:4290-4298.

Garcia JE, Wilksch PA, Spring G, Philip P, Dyer A. 2014. Charazterization of digital cameras for reflected ultraviolet photography; implications for qualitative and quantitative image analysis during forensic examination. J Forensic Sci. doi: 10.1111/1556-4029.12274

Hart NS. 2002. Vision in the peafowl (Aves: *Pavo cristatus*). J Exp Biol. 205:3925-3935.

Loew ER, Fleishman LJ, Foster RG, Provencio I. 2002. Visual pigments and oil droplets in diurnal lizards: a comparative study of Caribbean anoles. J Exp Biol. 205:927-938.

Párraga CA, Troscianko T, Tolhurst DJ. 2002. Spatiochromatic properties of natural images and human vision. Curr Biol. 12:483-487.

Pike TW. 2011. Using digital cameras to investigate animal colouration: estimating sensor sensitivity functions. Behav Ecol Sociobiol. 65:849-858.

Stevens M, Cuthill IC. 2006. Disruptive coloration, crypsis and edge detection in early visual processing. P Roy Soc Lond B Bio. 273:2141-2147.

Stevens M, Lown AE, Wood LE. 2014. Color change and camouflage in juvenile shore crabs *Carcinus maenas.* Front Ecol Evol. doi: 10.3389/fevo.2014.00014.

Stevens M, Párraga CA, Cuthill IC, Partridge JC, Troscianko TS. 2007. Using digital photography to study animal coloration. Biol J Linn Soc. 90:211-237.

Westland S, Ripamonti C. 2004. Computational colour science using MATLAB. Chichester, UK: John Wiley & Sons, Ltd.
